# Chondroitin sulfate proteoglycan 4 specific chimeric antigen receptor therapy for pediatric solid tumors

**DOI:** 10.1186/2051-1426-2-S3-P39

**Published:** 2014-11-06

**Authors:** Nicholas Tschernia, Rimas Orentas, Crystal Mackall

**Affiliations:** 1 Pediatric Oncology Branch / National Cancer Institute, Henderson, United States; 2 Pediatric Oncology Branch, CCR, NCI, NIH, Bethesda, MD, USA, Bethesda, United States

## 

Chondroitin sulfate proteoglycan 4 (CSPG4) is a membrane bound proteoglycan, composed of an N-linked 280kDa glycoprotein, and a single 450kDa chondroitin sulfate linked proteoglycan [1]. CSPG4 expression has been documented on adult malignancies (melanoma, breast, mesothelioma) but has yet to be assessed on pediatric malignancies [2,3]. Furthermore, both monoclonal antibodies and chimeric antigen receptor (CAR) T cells against CSPG4 have shown modest anti-tumor efficacy in adult malignancies [2,3]. We screened a spectrum of pediatric solid tumor lines for CSPG4 expression to determine potential targets, then developed CSPG4-redirected CARs to target CSPG4+ pediatric solid tumors.

16 pediatric tumor lines were assessed for CSPG4 expression. CSPG4 was detected by flow cytometry in 69% of the cell lines assessed: osteosarcomas: 143b,HOS-MNNG,MG63; synovial sarcomas: SYO-I,HSSY-II, alveolar/embryonal rhabdomyosarcomas: Rh18c,Rh30/Rh36, medulloblastoma: DAOY, melanoma: Mel624,Mel1300. CSPG4 expression site density was estimated with BD PE Quantibrite beads by flow cytometry.

Using two different single chain variable fragments (scFv) known to target CSPG4 (225.28 and TP41.2), two anti-CSPG4 second generation CAR constructs were synthesized by transient transfection of retroviral packaging lines. Both constructs have identical intracellular signaling components (CD28, CD3z). Healthy donor T cells were transduced with viral supernatants, and assessed for CAR expression. Anti-tumor activity was measured by cytokine release and tumor specific lysis. Cell surface expression of the CSPG4-based CARs was detected by flow cytometry using conjugated Protein L to target the expressed scFv light chain. 225.28 and TP41.2 based CAR T cells induced 25% and 31% lysis of target pediatric tumor lines, respectively (E:T ratio of 20:1). On further investigation, TP41.2 based CAR T cells were able to specifically lyse CSPG+ cell lines 143b, Rh30, and Rh18c at 40%, 37%, and 16%, respectively (E:T ratio of 30:1). Additionally, the TP41.2 and 225.28 CAR T cells were assessed for Interferon gamma (IFNy) production by flow cytometry. Compared to mock T cells coincubated with varying tumor lines (143b, Rh30, DND41), the TP41.2 and 225.28 CAR+ T cells had significantly positive populations for IFNy production (p < 0.0001), predominantly in the CD8+ population.

High levels of CSPG4 are present on a variety of pediatric solid tumors. TP41.2 CSPG4-based CAR T cells target, generate significant levels of IFNy, and lyse osteosarcoma *in vitro *at significant levels (p = 0.01). Targeting CSPG4 with CAR redirected T cells represents a new strategy for eliminating these pediatric malignancies in an antigen specific, cell-mediated manner.

## References

[B1] Burns WR Zhao Y Frankel TL A high molecular weight melanoma-associated antigen-specific chimeric antigen receptor redirects lymphocytes to target human melanomas Cancer Res 2010 70 8 3027 33 10.1158/0008-5472.CAN-09-2824 20395199 PMC3245576

[B2] Geldres C Savoldo B Hoyos V T Lymphocytes Redirected against the Chondroitin Sulfate Proteoglycan-4 Control the Growth of Multiple Solid Tumors both In Vitro and In Vivo Clin Cancer Res 2014 20 4 962 71 10.1158/1078-0432.CCR-13-2218 24334762 PMC3944408

[B3] Campoli MR Chang CC Kageshita T Human high molecular weight-melanoma-associated antigen (HMW-MAA): a melanoma cell surface chondroitin sulfate proteoglycan (MSCP) with biological and clinical significance Crit Rev Immunol 2004 24 4 267 96 10.1615/CritRevImmunol.v24.i4.40 15588226

